# Preliminary Study of Microbial Corrosion of Stainless Steel AISI 304 Under Conditions Simulating Deep Radioactive Waste Disposal

**DOI:** 10.3390/ma18235329

**Published:** 2025-11-26

**Authors:** Elena Abramova, Oleg Tripachev, Natalia Shapagina, Alexey Safonov

**Affiliations:** Frumkin Institute of Physical Chemistry and Electrochemistry Russian Academy of Sciences (IPCE RAS), 31-4, Leninsky Prospect, 119071 Moscow, Russia; gorchicta246@mail.ru (E.A.); tripachevov@mail.ru (O.T.); fuchsia32@bk.ru (N.S.)

**Keywords:** stainless steel AISI 304, DGR geochemical conditions, biogenic and biogenically mediated corrosion, biofilms, microbial metabolites, potentiodynamic polarization curves

## Abstract

This work involved the laboratory modeling of biogenic and biogenically mediated corrosion of AISI 304 stainless steel under geochemical conditions representative of the geological disposal of radioactive waste at the Yeniseisky site (Russia). Experiments with a single glucose stimulation of a microbial community sampled from a depth of 450 m established that the initial dominance of organotrophic microflora (primarily genera such as *Xanthobacterium*, *Novosphingobium*, *Hydrogenophaga*, and *Pseudomonas*) during the first stage (up to 30 days) led to the formation of a microbial biofilm. This biofilm resulted in uniform surface corrosion at a rate of up to 16 µm/year, which is more than 30 times higher than the corrosion rate in the abiotic control. This acceleration is attributed to the accumulation of microbial metabolites, including acetate, ethanol, formate, succinate, n-butyrate, and lactate. The subsequent development of chemotrophic iron- and sulfur-cycling microflora (dominated by genera such as *Sideroxydans*, *Pseudomonas*, *Geobacter*, *Desulfuromonas*, *Desulfovibrio*, and *Desulfomicrobium*) during the second stage of microbial succession (days 60–120) led to the formation of a pit density 10 times greater than that in the abiotic control. It is important to note that the maximum corrosion rates and pit densities were observed upon the addition of a mixture of glucose and sulfate. An assessment of the role of various microbial metabolites and medium components using the potentiodynamic method demonstrated that the combined presence of hydrocarbonate, sulfide, and microbial metabolites in the solution caused a more than fivefold increase in the corrosion current. Thus, the results demonstrate the complex nature of corrosion processes under conditions modeling the geological disposal of radioactive waste, where biological and abiotic factors interact, creating a synergistic effect that significantly enhances corrosion.

## 1. Introduction

Today, the safest way to handle radioactive waste (RW) and spent nuclear fuel (SNF) is to place them in deep geological formations using an engineered safety barrier (ESB) system [1,2]. In countries with a closed nuclear fuel cycle, such as Russia, it is planned to use glass matrices placed in a metal container for the disposal of high- and intermediate-level waste. Contact of the container with the geological environment should be prevented by a buffer barrier material based on bentonite clay, which has high anti-filtration properties [3]. In addition, the container material must have high corrosion resistance in an aggressive water environment. This requirement is one of the key ones to ensure long-term safety and prevent radionuclides from entering the geological environment. The following materials are considered for containers with radioactive waste [4,5]: carbon steel, stainless steel, copper, alloys based on nickel, palladium, and silicon carbide. Stainless steel as a material for containers for radioactive waste matrices is considered in France, Switzerland, and Belgium [6].

Stainless steels generally have good corrosion resistance due to the formation of a protective passive chromium oxide layer [7,8]. However, the passive layer is destroyed in an environment containing corrosion activator ions such as Cl-, Br-, I-, etc. [9,10,11], since halide ions are strong electron donors and are capable of participating in the oxidation of a number of metals, including iron [12,13]. Most often, stainless steels are subject to localized corrosion destruction, in particular, pitting corrosion, which causes barely noticeable, point, deep, and sometimes through damage on the metal surface [7,14,15,16,17,18]. Since the passive layer on the metal surface is not a homogeneous system, pitting occurs due to the presence of anodic and cathodic areas. Most often, in the resulting galvanic pair, the pitting acts as the anode, and the rest of the metal surface acts as the cathode. At the anode, electrons are released, which reduces oxygen on the cathode (passivated) part of the metal. Another dangerous type of localized dissolution of stainless steels is intercrystalline corrosion, which occurs in chromium-depleted zones due to the formation of chromium carbide during slow cooling of the steel, which in turn causes a potential difference at the grain boundary and in its matrix [19]. As a result, the chromium content near the grain boundaries is lower than on the rest of the surface, making them less passivated. As a result, such chromium-depleted areas are the anode, and the grain matrix is the cathode. Another type of localized corrosion of stainless steels is stress corrosion cracking [13], arising from the simultaneous action of an aggressive corrosive environment and tensile stresses on a metal surface [13]. As a result, defects in the form of pits and cracks appear on the stretched sections of the metal surface, which increase local stresses in the metal and are also a source of H+, according to the reaction Cr^3+^ + 3H_2_O → Cr(OH)_3_ + 3H^+^ [20]. Such defects act as anodes in relation to the unstretched part.

It is important to note that microbial processes can be the initiators of corrosion of stainless steels. In this case, the corrosion rate will be determined by the synergistic nature of biological and abiotic reactions [21,22]. Microorganisms participate in the corrosion of stainless steel through the destruction of the passive layer on the surface [23,24]. Chromium-deficient zones create micro-roughness on the surface, thereby creating preferential sites for bacterial attachment and biofilm development [25]. Further microbial-induced corrosion (MIC) in biofilm is a combination of chemical and electrochemical processes [26,27]. It involves substances produced by microorganisms, such as organic acids or hydrogen sulfide [28], as well as the processes of electron consumption from metal and stimulated by extracellular electron transfer [29,30,31]. In [32], which investigated microbial corrosion of stainless steels of different compositions, the important role of bacteria of the *Beggiatoaceae* family, iron-oxidizing bacteria, *Candidatus Tenderia* sp., etc., was noted. As noted in a previous review [33], surface hydrophobicity and the composition of alloying elements exert a strong influence on bacterial attachment. The presence of nickel demonstrates a dual role: it has an inherent ability to facilitate bacterial adhesion, yet concurrently, both nickel and chromium can mitigate material biofouling owing to their toxic properties. To achieve antibacterial properties, several researchers have suggested alloying steel with elements such as Mo, Ru, Cu, and Ag [34]. Through the formation of insoluble and bacteriotoxic oxide films on the surface of stainless steel, MIC predominantly manifests as pitting corrosion, a characteristic also observed in abiotic environments [35]. It is important to note that most studies on stainless steel corrosion have utilized model pure cultures of microorganisms. Undoubtedly, working with pure cultures provides fundamental data on corrosion mechanisms and eliminates uncertainties associated with fluctuating metabolite composition, the complex formation of multi-species biofilms, and variations in the biogeochemical potential of a microbial community.

However, assessing corrosion processes under conditions that approximate natural environments—taking into account relevant geochemical parameters and the dynamic succession of the microbial community—is a crucial next step for advancing corrosion models from a single-species to a community-level perspective.

Understanding the processes and mechanisms of material evolution occurring in the deep geological repository (DGR) is extremely important when creating long-term safety models for the repository. Evaluation of the MIC of materials is one of the important aspects in assessing the long-term safety of geological disposal of medium-level waste (MLW) and high-level waste (HLW) [21,36]. In Russia, the possibility of creating DGR in the Krasnoyarsk Territory in a granitoid massif is being considered [37,38].

Our previous studies [39,40] demonstrated that the subsurface microflora at the Yeniseisky site can promote the corrosion of carbon steel and copper through the production of hydrogen sulfide, methane, and hydrogen. This work aims to evaluate the role of this underground microflora in the localized corrosion of AISI 304 stainless steel, a candidate container material, and to identify the key mechanisms of bio-induced corrosion.

## 2. Materials and Methods

### 2.1. Stainless Steel Biocorrosion by Microbial Community

Rectangular specimens (10 × 15 × 1 mm) of AISI 304 stainless steel were used, with a nominal composition of (wt. %): 0.8 C, 0.8 Si, 0.2 Mn, 0.04 P, 0.02 S, 18 Cr, 9 Ni, 0.3 Cu, 0.4 Ti, balance Fe. Sample preparation involved ultrasonic cleaning for 25 min in a 1:1 (volume-to-volume) ethanol–toluene mixture. To establish an anaerobic environment for the experiments, the samples were immersed in 50 mL of liquid medium in sealed vessels. The headspace of the vessels was evacuated and subsequently backfilled with pure argon (100%) to a volume of 50 mL.

Solution (×10^−4^ mol/L: K^+^—1.15, Mg^2+^—4.95, Ca^2+^—12.2, Na^+^—8.87, Cl^−^—25.6, SO_4_^2−^—4.95, HCO_3_^−^—8.87; pH 7.0; t = 20 ± 1 °C; total dissolved solids (TDSs) = 278.2 mg/L) modeling water from the Yeniseisky site (Russia), where construction of DGR is planned, was used as the liquid phase [41,42].

Groundwater from well R-8 (mg/L) (Mg^2+^—11.1, Ca^2+^—11.3, K_+_—2.1, SO_4_^2−^—1.51, Cl^−^—8.9, HCO_3_^−^—173.8, NO_3_^−^—3.0, CO_3_^2−^—10.5, Na^+^—52.6, pH 7.8, t = 20 ± 1 °C, TDS = 274.8 mg/L) was used as a source of underground microbiota [39]. Groundwater made up 1/10 of the model solution.

To stimulate the growth of microbiota, H_2_ (100%, extra pure) was added to the gas phase (Sample MWH-bio), glucose 1 g/L (Sample MWG-bio), glucose 1 g/L and sodium sulfate 1 g/L (Sample MWGS-bio). A solution of model water (Sample MW0) and a solution of model water with the addition (1/10) of underground water R-8 (Sample MW-bio) were used as a control. The experimental scheme is given in Table 1.

### 2.2. The Effect of Microbial Components on Stainless Steel Corrosion

The corrosion–electrochemical studies of steel were carried out using the potentiodynamic method. The intersection points of the Tafel sections of the anodic and cathodic processes (linear sections on the curves starting at a distance of 50 ÷ 100 mV in both directions from the corrosion potential) were determined from the cathodic and anodic potentiodynamic curves E_corr_). The intersection point corresponds to the corrosion potential E_corr_ with the corresponding corrosion current I_corr_. The corrosion rate was calculated using the Stern–Geary equation [43]. The measurements were carried out similarly to those described in [38,39], using a standard three-electrode electrochemical cell with separated spaces of the auxiliary electrode and the reference electrode. A disk electrode, a metal cylinder made of AISI 304 steel with a diameter of 5 mm, pressed into a Teflon housing, was used as the working electrode. The free surface was a disk with an area of 0.196 cm^2^. Before each experiment, the AISI 304 surface was brought to a mirror finish by processing with abrasives of different sizes (1200 ÷ 600 grit). Then, the electrode was thoroughly washed with deionized water and placed in the working cell. A platinum electrode was used as the auxiliary electrode, and a silver chloride electrode was used as the reference electrode [44,45].

Before the experiment, the solution was purged with high-purity argon (99.998%) for half an hour, and then the working electrode was placed in the cell, and the solution was purged for another 1.5 h to establish a steady-state potential. After that, polarization curves were measured in the potentiodynamic mode with a sweep rate of 1 mV/s separately in the cathode and anodic sides, waiting for the steady-state potential to be established before each measurement.

Solutions based on natural water with a known quantitative and qualitative content of salts were used as working solutions, with the addition of 0.01 M NaClO_4_ to increase the electrical conductivity of the solution. Components were also added to the solution, as indicated in Table 1. The experiments used the main macrocomponents of groundwater, typical for the Yeniseisky site, as well as possible components that can form as a result of microbial metabolism. To assess the role of concentrations of the main components, a model solution was used, calculated on the basis of the composition of real samples in which the concentrations of one of the components were increased The growth of the corrosion current is directly proportional to the growth of the annual corrosion rate and serves as a reliable criterion for assessing the stability of metals in corrosive environments.

### 2.3. Methods

**Evaluation of Biofouling and Metabolic Activity.** The extent of biofouling was evaluated via the MTT test, which measures cellular respiratory activity [39,46]. The assay was conducted on both the material surfaces and the surrounding liquid medium. The resulting optical density values for reduced formazan, indicative of metabolic activity, are reported per cm^2^ of sample surface area.

**Quantification of Volatile Fatty Acids.** Volatile fatty acid (VFA) concentrations were analyzed by gas chromatography (GC) on a Crystal 5000.2 system (Chromatec, Moscow, Russia). The GC was configured with a flame ionization detector and a ZB-WAXplus capillary column (30 m × 0.25 mm, 0.35 µm film thickness; Phenomenex (Torrance, CA, USA). The oven temperature was programmed from 100 °C to 180 °C with a 10 °C min^−1^ ramp. High-purity nitrogen served as the carrier gas.

**Optical ex situ microscopy.** Optical microscopy was used to establish the nature of dissolution of the metals under study after their exposure to corrosive environments. For this purpose, a Biomed PR-3 optical microscope (Biomed Service LLC, Moscow, Russia), with magnification 50× and 100×, was used. The depth of local defects (pits) was determined using the Neophot-2 metallographic optical microscope (Carl Zeiss, Jena, Germany). The average maximum depth of defects was determined from the values obtained for the three deepest pits [47].

**Scanning electron microscopy (SEM).** Microstructural analysis was conducted with a TESCAN MIRA3 FEG-SEM device (Joint Use Center, Vernadsky Institute, RAS Moscow, Russia). The examinations were carried out at an accelerating voltage of 20 kV, utilizing both secondary (SE) and backscattered electron (BSE) detection modes to assess surface topography and compositional contrast.

**Measurement of corrosion rate by the gravimetric method.** The corrosion rate was quantified using the gravimetric (weight-loss) method. After exposure, the samples were chemically cleaned in a 0.5 M nitric acid solution for 60 min at room temperature to strip corrosion products, allowing for the accurate measurement of metal loss. The corrosion rate of the samples (μm/year) was calculated using Equation (1) [48]:

(1) $$V_{corr}=\frac{∆m\cdot 8760}{S\cdot t\cdot \rho }$$

where Δm—average mass loss of metal specimens after corrosion tests, g; S—surface area of metal specimens, m^2^; t—test time, h; ρ—density of metal, g/cm^3^. The corrosion rate is expressed in µm/year.

**Measurement of corrosion rate by the potentiodynamic method.** Potentiodynamic curves and corrosion potential were measured using a potentiostat Solartron SI 1287 (Solartron Analytical, Farnborough, UK). Measurements were carried out using a standard three-electrode cell.

**X-ray photoelectron spectroscopy.** Chemical analysis of the composition of surface layers formed on metals during their exposure to corrosive environments was carried out using XPS. The XPS spectra of the surface layers were recorded on an OMICRON ESCA+ spectrometer (OMICRON, Rodgau-Dudenhofen, Germany). The pressure in the analyzer chamber was maintained at no more than 8 × 10^−10^ mbar, and the radiation source was an Al anode (AlKα 1486.6 eV). The analyzer transmission energy was 20 eV. To account for the charging of the samples, the position of the XPS peaks was standardized by the C1s peak of hydrocarbon contaminants (E_b_ = 285.0 eV). The spectra were decomposed into components using the UNIFIT 2009 program. The background was determined using the Shirley method.

For the analysis of microbial diversity, total genomic DNA was isolated using the ZymoBIOMICS™ DNA Mini-prep Kit (Zymo Research, Tustin, CA, USA). Hypervariable regions of the 16S rRNA gene were targeted for amplification during library preparation: the V3-V4 region was amplified using the degenerate primers For341 and Rev806, and the V4 region was amplified using primers For515 and Rev806. Amplification was performed by real-time PCR on a CFX96 Touch thermocycler (Bio-Rad, Hercules, CA, USA) with qPCR mix-HS SYBR (Evrogen, Moscow Russia). The thermal cycling profiles for the V3-V4 and V4 regions differed in their annealing temperatures (54 °C and 58 °C, respectively), while denaturation and elongation steps were conducted at 96 °C and 72 °C for both. The resulting amplicons were purified using Agencourt AMPure XP magnetic beads (Beckman Coulter, Brea, CA, USA). Finally, high-throughput sequencing of the prepared libraries was performed on a MiSeq platform (Illumina, San Diego, CA USA) using a MiSeq Reagent Kit v2 (500 cycles).

## 3. Results

### 3.1. Dynamics in Microbial Community Structure

**Figure 1:**
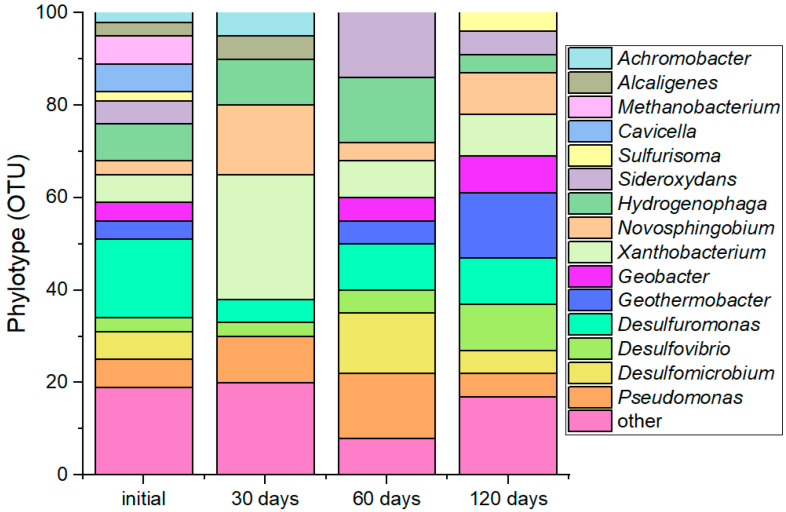
Dynamics in microbial community structure on days 30, 60, and 120 in the MWGS-bio experiment.

Figure 1 presents data on the changes in the microbial community structure over the course of the groundwater experiment with a single injection of glucose and sulfate (MWGS-bio). Under the experimental conditions, a shift in the microbial community was observed by day 30, characterized by the dominance of organotrophic anaerobic bacteria. This shift, induced by the glucose addition, was marked by the prevalence of genera such as *Xanthobacterium* (up to 25% of OTUs), *Novosphingobium* (up to 15% of OTUs), *Hydrogenophaga*, and *Pseudomonas* (up to 10% of OTUs), as well as *Alcaligenes and Achromobacter* (5% of OTUs each). Most of these organisms are capable of fermenting saccharides, producing various organic metabolites.

By day 60, following the depletion of the initial carbon source, an increased contribution from bacteria involved in the iron cycle was noted in the microbial community. These included representatives of the genera *Sideroxydans* (up to 15% of OTUs), Pseudomonas (up to 15% of OTUs), *Geobacter*, and *Geothermobacter* (5% of OTUs each). Furthermore, an increased contribution was observed from sulfur-cycling bacteria belonging to the genera *Desulfuromonas*, *Desulfovibrio*, and *Desulfomicrobium*.

By day 120, the community was dominated by sulfur-cycling bacteria: *Desulfuromonas* (up to 10% of OTUs), Desulfovibrio (up to 10% of OTUs), *Desulfomicrobium*, and *Sulfurisoma* (up to 5% of OTUs). The relative abundance of organotrophic bacteria from the genera *Xanthobacterium*, *Novosphingobium* (up to 15% combined), *Hydrogenophaga*, and Pseudomonas did not exceed 5% of OTUs for each taxon.

### 3.2. Microbial Fouling of Samples

**Figure 2:**
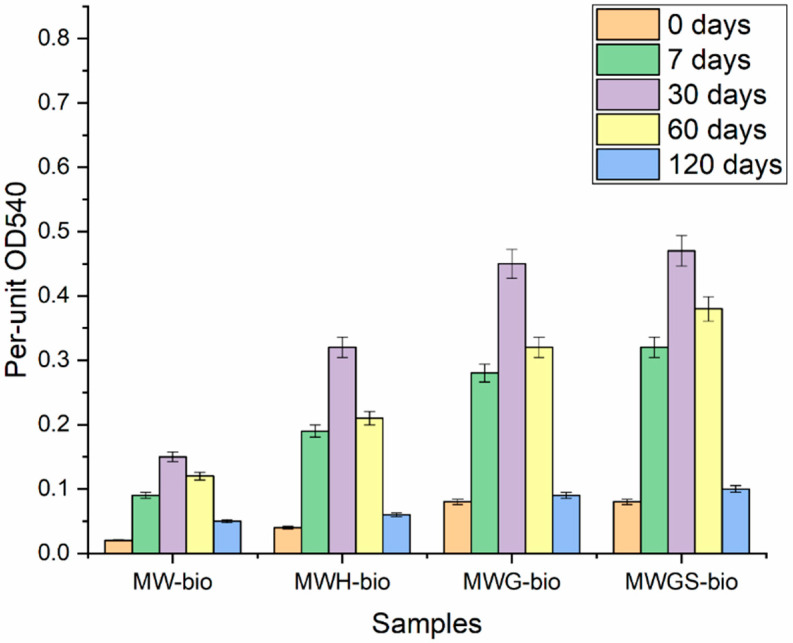
Respiratory activity of biofilms on the surface of 1 cm^2^ of samples at 0, 7, 30, 60, and 120 days.

According to the results of respiratory activity assessment on the surface of stainless steel samples (Figure 2), the maximum growth of biofilms was observed on the 30th day of the experiment. The maximum values of microbial activity were found in samples in which growth was stimulated by glucose (MWG-bio) and glucose with sulfate ion (MWGS-bio). When adding hydrogen as an electron donor (MWH-bio), microbial activity was 30 ÷ 40% higher than when stimulated by glucose. A significant decrease in activity was noted on the 120th day; no additional stimulation was performed.

Based on the microbial diversity assessment data from the MWGS-bio experiment presented in the previous section, it can be concluded that the addition of glucose leads to the activation of organotrophic biofilm-forming bacteria between days 7 and 30. Subsequently, following the depletion of the carbon source, a gradual decline in the activity of the organotrophic microflora is observed. From days 60 to 120, the microbial community is predominantly composed of sulfur- and iron-cycling bacteria, whose activity may contribute to biofilm degradation under conditions of limited available organic matter.

### 3.3. Volatile Acids and H_2_S Formation

Previous investigations have characterized the microbial community composition, revealing the presence of aerobic and anaerobic organotrophic bacteria, as well as bacteria involved in iron and sulfur cycling, with a predominance of sulfate-reducing bacteria [32]. To elucidate the role of organic metabolites in the corrosion process, their qualitative and quantitative profiles were assessed at various stages of microbial community development.

**Figure 3:**
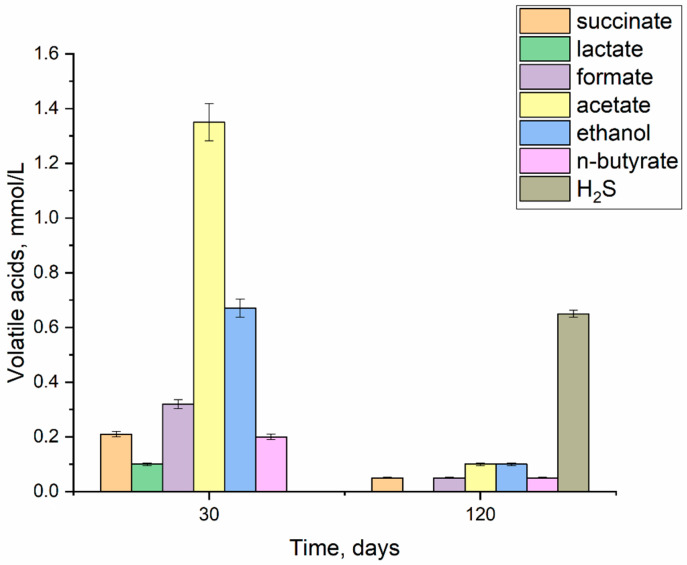
Content of volatile organic compounds and H_2_S when stimulating the growth of microbiota with glucose (1 g/L) on days 30 and 120 (in the MWGS-bio experiment).

On the 30th day, the glucose-stimulated sample showed accumulation of volatile microbial metabolites, mmol/L: succinate and n-butyrate—0.2, lactate—0.1, formate—0.32, acetate—1.35, and ethanol—0.67. By the 120th day of the experiment, the concentration of organic metabolites decreased by 90%, indicating their consumption as a result of microbial succession (Figure 3). The effect of microbial metabolites on stainless steel corrosion is possible only in the early stages (up to 30 days), when the accumulation of organic compounds is maximum. On the 120th day, the concentration of hydrogen sulfide was measured at 0.65 mmol/L. The accumulation of microbial metabolites during fermentation facilitated the proliferation of sulfate-reducing bacteria.

**Figure 4:**
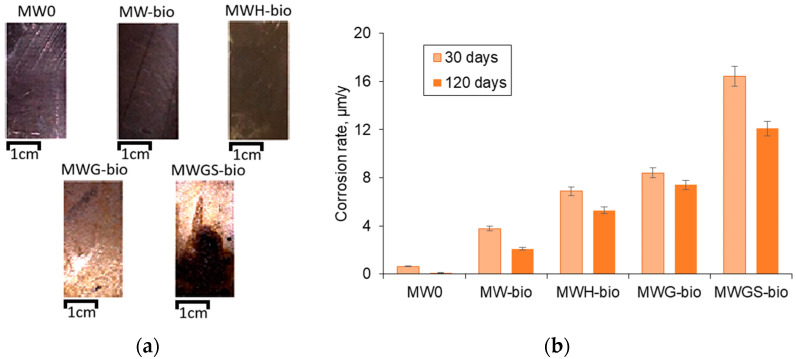
Gravimetric tests: (**a**) surface of plates on the 120th day of the experiment; (**b**) maximum corrosion rate of stainless steel on the 30th and 120th day (calculated using Equation (1)).

During the gravimetric tests, general etching of the stainless steel surface was recorded under biological influence (Figure 4a). When stimulating the growth of microbiota with glucose and sulfate ions (MWG-bio and MWGS-bio), significant damage to the surface was observed, and local foci in the form of pits and ulcers were noted. In low-mineralized model water, the surface did not change visually. The corrosion rate was calculated using Equation (1). According to the results of gravimetric tests, the maximum corrosion rate was noted on the 30th day (Figure 4b), which is consistent with the data on microbial fouling (Figure 1). The corrosion rate with the addition of underground biota was 5 times higher compared to the control sample. Stimulating the growth of biota with hydrogen (MWH-bio) and glucose (MWG-bio) led to a 2-fold increase in corrosion processes. The maximum value was noted for samples with the addition of glucose and sulfate ions (MWGS-bio), and the corrosion rate was 16 μm/year. On day 120, a decrease in the corrosion rate by 10–20% was noted.

### 3.4. Description of Defects on the Surface of Stainless Steel

**Figure 5:**
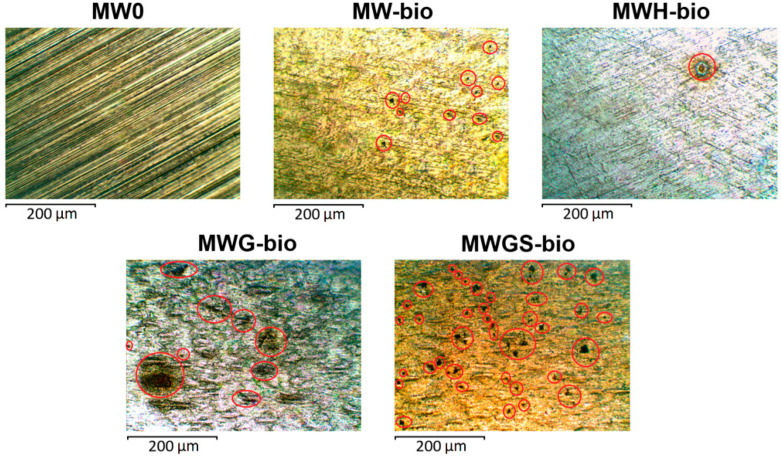
Micrographs of the surface of stainless steel samples incubated under various conditions for 120 days. Pitting is marked with red circles.

Figure 5 shows the results of corrosion tests of stainless steel samples exposed to different environments for 120 days. No defects were found on the surface of the model water (MW0). When underground microbiota (MW-bio) was added, localized metal dissolution foci were observed. The average number of defects (N_average_) per 1 mm^2^ was 6, and the average diameter (d_average_) of the defects was 13.8 μm. When hydrogen (MWH-bio) was added to the gas phase, the number of defects N_average_ = 1 with d_average_ = 26.7 μm. Stimulation of the biota with glucose increased the number of defects to 13 with an average diameter of 18.8 μm. The presence of glucose and sulfate ions stimulated the growth of sulfate-reducing bacteria, which led to the formation of pitting colonies on the surface of stainless steel N_average_ = 63, and d_average_ = 16.1 μm.

Taking into account the local nature of corrosion damage on stainless steel, as well as data on the average size and average number of point defects (Table 2), the corrosion rate was recalculated for the average pitting depth. According to the calculated data, the greatest pitting depth was recorded for samples MWH-bio and MW-bio, at 88 µm and 83 µm, respectively. The smallest depth was noted for the MWGS-bio sample, where the growth of microbiota was stimulated by glucose and sulfate.

**Figure 6:**
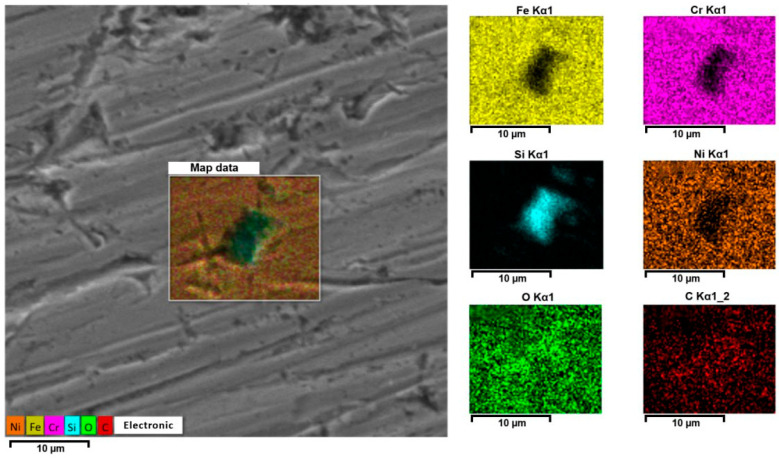
SEM image of stainless steel surface after biological testing with glucose stimulation for 120 days.

**Figure 7:**
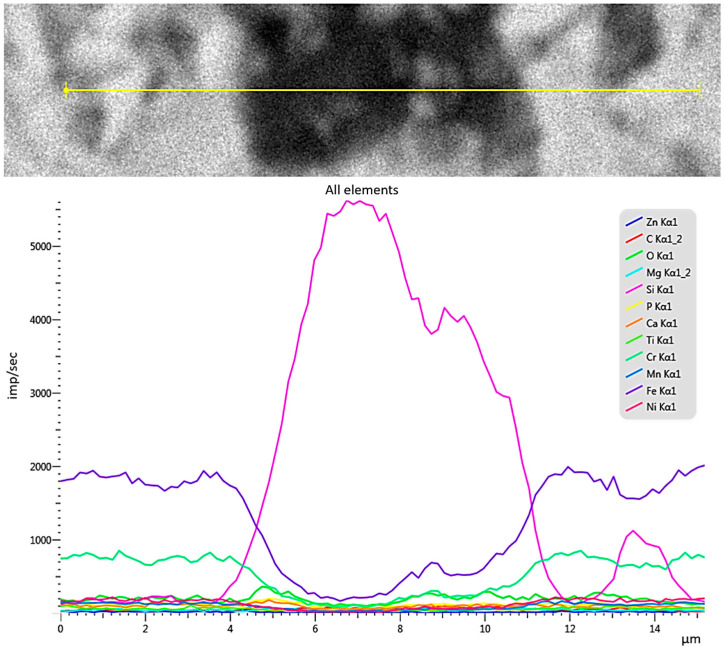
Distribution of elements in pitting.

Figure 6 shows a detailed map of the distribution of elements in the formed pit under biological influence in the presence of glucose for 120 days. Depletion of iron and chromium in the pitting area was observed. Carbon and oxygen are uniformly distributed on the metal surface and in the local corrosion focus. Most of the silicon is concentrated in the local focus itself (Figure 7).

This distribution of elements was observed across the entire surface of the specimen. Silicon–oxygen compounds in the form of silicon dioxide create weak points in the passive film, leading to localized rupture of the protective layer and the initiation of pitting corrosion. The presence of chloride and sulfate ions, as well as glucose, in the electrolyte further enhances the localized corrosive destruction of the stainless steel surface. In this case, pitting acts as the anode. The following processes occur in the anodic region: oxidation of the metal via the reaction Fe → Fe^2+^ + 2e^−^; interaction with chloride ions through the reaction Fe^2+^ + Cl^−^ → [FeCl]^+^; and formation of complexes with silicon dioxide. In the cathodic region, the processes include: oxygen reduction according to the reaction O_2_ + 2H_2_O + 4e^−^ → 4OH^−^; neutralization of acidic products; and formation of a protective layer [49,50]. The formation of silicon dioxide facilitates the penetration of chloride ions, thereby accelerating corrosion in the anodic zone. As a result, there is an intensification of localized stainless steel degradation and a reduction in its overall corrosion resistance (Figure 7).

**Figure 8:**
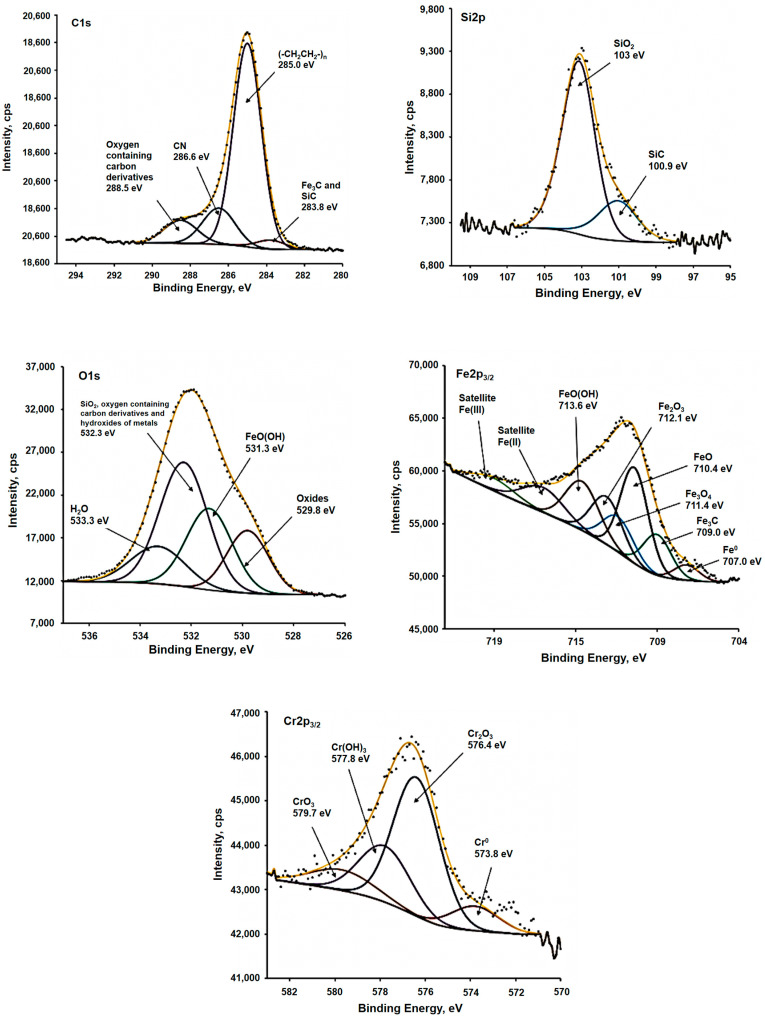
XPS spectra of the stainless steel surface after corrosion tests in the presence of underground microbiota stimulated by glucose for 120 days.

Also, the surface of stainless steel after corrosion tests in the presence of underground microbiota was assessed using XPS. According to the overview spectrum of the surface in the binding energy range of 1100 ÷ 0 eV, sodium and oxygen lines, including their Auger lines, as well as lines of iron, chromium, calcium, silicon, and nitrogen, and hydrocarbon contaminants are observed on the surface of the sample before corrosion tests (Figure 8). The complex spectrum of Fe2p3/2 electrons can be decomposed into 8 components: metallic iron Fe^0^ (707.2 eV), iron carbide with a binding energy of 709 eV, as well as oxides: Fe_3_O_4_ (711.4 eV), FeO (710.4 eV) and Fe_2_O_3_ (712.1 eV), and the peak of metahydroxide FeO(OH) with a maximum of 713.6 eV, as well as characteristic satellites for Fe(II) and Fe(III). Chromium, which is part of the alloy, is in metallic Cr^0^ (573.8 eV), Cr_2_O_3_ (576.4 eV), CrO_3_ (579.7 eV), and also in hydroxide Cr(OH)_3_ (577.8 eV) states. The spectrum of silicon Si2p can be divided into two doublets related to SiC (100.9 eV) and silicon dioxide with a binding energy of 103 eV [51,52,53,54,55].

Iron is in the same state as the sample before the corrosion studies, with the exception of Fe^0^. No chromium was detected, which may be due to both the depletion of the surface layer of this element and the increase in the thickness of the iron oxide layer and the layer of corrosion products. A shift in the peaks to the region of higher values by about 0.2 eV is also observed, which can also be explained by an increase in the thickness of the surface layers and the loss of energy in these layers. According to the spectrum of iron Fe2p3/2 electrons, an additional state of FeSO_4_ with a maximum of 714.6 eV was observed. On the spectrum of S2p electrons, three states of FeS (162.3 eV), CS_2_ (163.5 eV), and a doublet in the region of high energies of FeSO_4_ (168.5 eV) were observed. At the same time, chromium was detected on this sample and, according to the spectrum of Cr2p3/2 electrons, it was in Cr_2_O_3_ (576.5 eV), CrO_3_ (579.8 eV), and also in the hydroxide Cr(OH)_3_ (577.8 eV) states [51,52,53,54,55].

## 4. Discussion

The most pronounced MIC effect was observed on AISI 304 stainless steel upon contact with a subsurface microbial community sampled from the prospective site of a future radioactive waste repository. This was demonstrated in an experiment where the community was stimulated with organic matter (e.g., glucose) and sulfates (the MWGS-bio experiment). Under these conditions, the corrosion rate reached 16 µm/year by day 30, exceeding the corrosion rate in the abiotic control by more than 30-fold.

By day 30, maximum biofilm formation was observed on the specimens, a characteristic feature across all experiments. Glucose stimulation led to the development of organotrophic microflora with a fermentative metabolism under anaerobic conditions (primarily genera *Xanthobacterium*, *Novosphingobium*, *Hydrogenophaga*, and *Pseudomonas*). Most of these organisms are capable of fermenting saccharides, producing various organic metabolites [56] and forming biofilms [57]. Significantly, the genera detected in our work are known from the literature to be involved in steel corrosion [15,58,59,60].

Under the experimental conditions, the following characteristic fermentation products were detected in the liquid phase by day 30 (mmol/L): succinate and n-butyrate—0.2, lactate—0.1, formate—0.32, acetate—1.35, and ethanol—0.67. During this initial stage (up to 30 days), the corrosion was predominantly uniform, associated with the dissolution of the passivating chromium and nickel oxides characteristic of AISI 304 steel, which has a composition of 18 wt% Cr, 9 wt% Ni, and 0.3 wt% Cu. Thus, based on the data on microbial respiration occurring on the steel surface, no significant toxic effect of the steel composition on the multi-component microbial community was detected [40].

In our previous work [40], we published data on copper corrosion using the same microbial community. The findings demonstrated that this community is capable of forming biofilms and inducing copper corrosion, with a maximum corrosion rate reaching 9.8 µm/year. In experiments [40] with microbial community growth stimulated by glucose, the development of fermentative and biofilm-forming microorganisms (*Xanthobacteraceae*, *Hydrogenophaga*) was observed by day 20, while sulfur cycle bacteria (*Desulfuromonas*, *Desulfomicrobium*) were detected by day 90.

However, dissolved copper is known to exert a significant toxic effect on microorganisms at concentrations exceeding 250 mg/L. The observed biofilm formation and concomitant corrosion despite this toxicity may be associated with the biofilm’s ability to sequester metals, thereby protecting the microbial cells from their inhibitory effect.

The role of biofilms in steel corrosion is well-established and is widely considered one of the most critical factors in microbiologically influenced corrosion (MIC). Bacterial attachment is recognized as a precursor step to microbial corrosion, significantly influencing the corrosion behavior of materials at the material–solution interface [61]. Several key mechanisms through which biofilms intensify MIC are known. The primary role of the biofilm is to create a distinct, aggressive chemical environment at the metal surface. It acts as a barrier, facilitating the formation of differential aeration cells and the accumulation of microbial metabolites (e.g., organic acids, sulfides). This locally alters pH and chemistry, disrupting the passive oxide layer [62]. Biofilms enable specific metabolic reactions that drive corrosion [63]: Electron Transfer (EMIC): Certain bacteria, like sulfate-reducing bacteria (SRB) and methanogens, can directly utilize elemental iron (Fe^0^) as an electron donor through extracellular electron transfer (EET), a mechanism heightened within conductive biofilms. Moreover, multi-species biofilms create synergistic consortia where the metabolic activity of one group supports another. For instance, facultative anaerobes can consume oxygen, creating an ideal anaerobic niche for SRB to thrive adjacent to the metal surface [64].

The processes occurring within the biofilm led to more profound corrosion effects, resulting in pit formation by days 60–120. We propose that this intensified corrosion is linked to a shift in the dominant microbial populations by day 60, marked by the prevalence of iron-cycling bacteria (dominated by genera such as *Sideroxydans*, *Pseudomonas*, and *Geobacter*), which are capable of dissolving iron corrosion products and chromium oxides [15].

By day 120, in the experiment with sulfate amendment, a substantial decrease in the contribution of organotrophic bacteria was observed, alongside the dominance of anaerobic sulfur-cycling bacteria from the genera *Desulfuromonas*, *Desulfovibrio*, and *Desulfomicrobium*. The formation of hydrogen sulfide (up to 0.6 mmol/L) by day 120 explains the maximum corrosion rate observed in the sulfate-amended experiment. Conversely, in the experiment without sulfate addition, no hydrogen sulfide production was detected, and the corrosion rate was two times lower than in the experiment with both glucose and sulfate.

It is important to note that in the experiment without amendments, the corrosion rate reached 4 µm/year. This corrosion is attributed to the involvement of chemical components characteristic of the future repository’s groundwater (e.g., hydrocarbonates, sulfates) in the microbial transformation of the steel. Even without the addition of external stimulants, biofilm formation was observed on the steel surface (Figure 2, MW-bio). In contrast, the corrosion rate in the synthetic water control without the microbial community was less than 1 µm/year.

As demonstrated in our study, stainless steel is characterized by localized breakdown of the passive film and autocatalytic propagation of pits. Under the model conditions, pit formation with depths ranging from 25 to 88 µm was observed over the 120-day experiment. In a previous study [57], it was found that microorganisms of the genus *Desulfovibrio* caused pitting with a depth of 4.8 μm within 7 days. In a comparable investigation [65], bacteria of the genus *Pseudomonas* induced pits reaching 7.4 μm in depth over a 14-day period.

To evaluate the specific role of biogenic and abiogenic environmental components, a model experiment was conducted using the electrochemical method.

**Figure 9:**
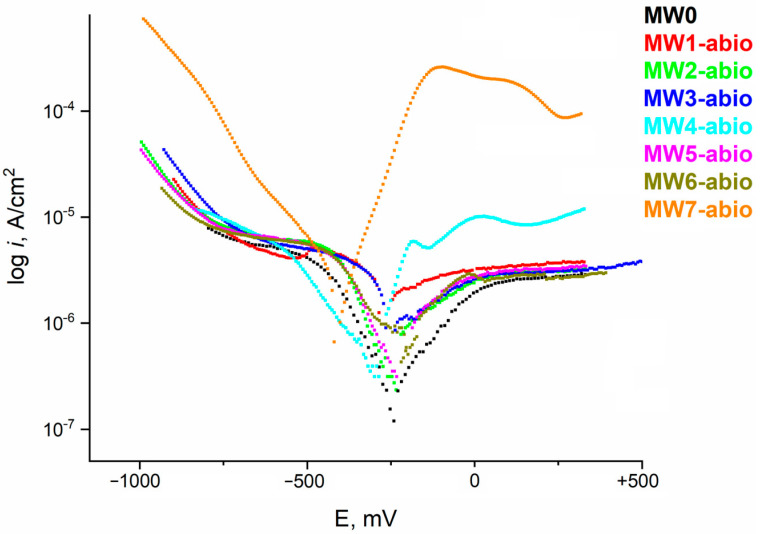
Potentiodynamic polarization curves of stainless steel electrodes in natural water with different steel additives.

Figure 9 shows the potentiodynamic curves obtained on a disk electrode made of AISI 304 steel in an inert atmosphere of natural water with various additives.

Potentiodynamic curves allow us to estimate the current density of anodic and cathodic processes on the metal surface. By finding the intersection point of the Tafel sections of anodic and cathodic processes, we can calculate the corrosion potential in various environments, as well as the corrosion current density, on the basis of which we can compare the average annual corrosion rates. The parameters of stainless steel in natural water were estimated in the presence of additives of various anions and microbial metabolites (containing organic acids and a number of other compounds) (Table 3).

The magnitude of the corrosion potential for different environments differs slightly. In the presence of sulfate anion, sulfide anion, and MW7-abio medium, a shift in the corrosion potential to the cathodic region is observed, not exceeding 75 mV. For other environments, this potential shift is less than 30 mV; a solution with the addition of microbial metabolites is characterized by a slight shift in E_corr_ to the anodic region of values. In general, the shift in E_corr_ to the cathodic side indicates more active corrosion of the metal in the presence of additives of the corresponding anions.

The corrosion current value is minimal for natural water with added background perchlorate anion; the current density is 0.233 μA/cm^2^. The minimal growth of the corrosion current is typical for additives of nitrate anion and sulfide anion. Microbial metabolites, sulfate, hydrocarbonate, and chloride anion contribute to a 2.5 ÷ 3-fold growth of the corrosion rate, and the presence of hydrocarbonate, sulfide, and microbial metabolites in the solution causes the maximum growth of the corrosion current, more than 5 times. Electrochemical data on the behavior of steel in various environments allow for a fairly quick assessment of the corrosion rate and are in good agreement with the data obtained earlier by other methods.

The analysis of polarization curves allows us to conclude that the addition of chloride, nitrate, sulfide, and microbial metabolites has little effect on the process of anodic dissolution of stainless steel with increasing overvoltage on the electrode. The addition of sulfate causes an increase in the anodic current at high overvoltages, while the addition of sulfide in the presence of microbial metabolites and hydrocarbonate anion causes a significant increase in the anodic current. To assess the stability of steel in these solutions, the values of the corrosion potential and current were found based on the intersection points of the Tafel sections of the cathodic and anodic processes (Table 1). The lowest corrosion current is characteristic of natural water with the addition of a background electrolyte. The addition of anions that promote specific processes on the steel surface—such as chlorides, sulfates, and hydrocarbonates—causes an increase in the corrosion current. Most likely, this effect is manifested in the acceleration of pitting corrosion, which makes the main contribution to the total corrosion current of steel. The maximum value of corrosion currents is characteristic of the MW7-abio environment, while it is worth noting that sulfide in the presence of only natural water and background does not cause a significant increase in the corrosion rate.

## 5. Conclusions

The use of AISI 304 stainless steel as a container material for the geological disposal of radioactive waste presents a complex trade-off. On the one hand, it is preferable due to its lower general corrosion rate compared to carbon steel. On the other hand, it carries the risk of localized pitting corrosion. This work presents initial experimental data on the durability of stainless steel under biogeochemical conditions that could develop upon water ingress into the container. A multi-component microbial community was shown to increase the corrosion rate from 0.4 µm/year in abiotic conditions to 4 µm/year without any stimulant addition, and up to 16 µm/year following a single amendment with glucose and sulfate.

The maximum rate of uniform corrosion was achieved during biofilm formation by predominantly organotrophic bacteria (genera such as *Xanthobacterium*, *Novosphingobium*, *Hydrogenophaga*, and *Pseudomonas*), driven by the production of biogenic acidic metabolites. In contrast, pit formation occurred at later stages and was associated with iron- and sulfur-cycling microorganisms (genera such as *Sideroxydans*, *Pseudomonas*, *Geobacter*, *Desulfuromonas*, *Desulfovibrio*, and *Desulfomicrobium*) that oxidize passive oxide films and produce hydrogen sulfide. Furthermore, potentiodynamic studies under model conditions identified chlorides and carbonates, alongside sulfide and microbial metabolites, as the most significant factors contributing to steel corrosion in the given geochemical environment. Thus, the results demonstrate the complex nature of corrosion processes under conditions modeling geological radioactive waste disposal, where biological and abiotic factors interact, creating a synergistic corrosive effect.

The findings of this study are primarily of a fundamental nature, as they do not fully simulate the various scenarios of groundwater contact with container materials following the long-term degradation or failure of engineered barriers. However, this research outlines the direction for future, more detailed investigations into the behavior of stainless steel in an underground research laboratory setting, should this material be selected as a candidate for radioactive waste containers.

Based on the conducted research, the following conclusions can be made:

1. It has been established that the local dissolution of stainless steel AISI 304 occurs under the influence of abiotic factors and the formation of bacterial biofilm. In the given conditions, the formation of a biological film on the surface of AISI 304 occurs much faster (within 30 days) than is usually observed in nature [66,67].
2. The maximum accumulation of microbial metabolites was experimentally recorded on day 30, including acetate, ethanol, formate, succinate and n-butyrate, and lactate. It was found that after 120 days, the concentration of organic metabolites decreases by 90%, which leads to a slowdown in the local dissolution of the stainless steel.
3. It was found that the presence of glucose and sulfate ions stimulates the growth of sulfate-reducing bacteria, which promotes the initiation of pitting corrosion and the formation of colonies of pits on the surface of stainless steel.
4. It has been shown that in anoxic environments, the passive film is unable to recover quickly after microbial exposure, and the metabolites enhance the locative destructive effect of chloride ions.
5. Analysis of the data from gravimetric measurements and electrochemical studies has shown the consistency of the results in assessing the corrosion behavior of steel under various conditions.

Thus, the results of the research demonstrate the complex nature of corrosion processes in the conditions of modeling the geological burial of radioactive waste, where biological and abiotic factors interact, creating a synergistic effect of corrosion.

## Figures and Tables

**Table 1:** Experimental scheme.

**1. Stainless Steel Biocorrosion by Microbial Community**			
**Conditions**	**Samples**	**Time, Days**	**Analyses**
− Model water (×10^−4^ mol/L): K^+^—1.15, Mg^2+^—4.95, Ca^2+^—12.2, Na^+^—8.87, Cl^−^—25.6, SO_4_^2−^—4.95, HCO_3_^−^—8.87; pH 7.0 pH 7.8; − Groundwater (R-8, with 1/10): (mg/L): Mg^2+^—11.1, Ca^2+^—11.3, K^+^—2.1, SO_4_^2−^—1.51, Cl^−^—8.9, HCO_3_^−^—173.8, NO_3_^−^—3.0, CO_3_^2−^—10.5, Na^+^—52.6, pH 7.8; − Plates of stainless steel AISI 304—10 × 15 × 1 mm; − Anaerobically (Ar, 100%), t = 20 °C	MW0-control—model water MW-bio—model water with 1/10 groundwater MWH-bio—H_2_ as a gas phase MWG-bio—glucose (1 g/L) MWGS-bio—glucose (1 g/L) + Na_2_SO_4_ (1 g/L)	7, 30, 60, 120	− Evaluation of Biofouling and Metabolic Activity (MTT test) − Quantification of Volatile Fatty Acids − Surface evaluation of stainless steel samples (visual evaluation) − Measurement of corrosion rate by the gravimetric method (calculated using Equation (1)) − X-ray photoelectron spectroscopy
**2. The Effect of Microbial Components on Stainless Steel Corrosion**			
**Conditions**	**Samples**	**Time, Days**	**Analyses**
− Model water with the addition − Stainless steel AISI 304 disc 5 mm diam − Anaerobically (Ar, 100%), t = 20 °C	MW0—model water * MW1-abio—NaCl (1 g/L) * MW2-abio—NaHCO_3_ (1 g/L) * MW3-abio—NaNO_3_ (1 g/L) * MW4-abio—Na_2_SO_4_ (1 g/L) * MW5-abio—Na_2_S (1 g/L) * MW6-abio—microbial metabolites * MW7-abio—microbial metabolites + Na_2_S (1 g/L) + NaHCO_3_ (1 g/L) *	3, 10, 20, 45, 90, 120	− Measurement of corrosion rate by electrochemical methods (potentiodynamic polarization curves), calculated using the Stern–Geary equation [43]

* When assessing the corrosion rate using electrochemical methods, an additive of 0.01 M NaClO_4_ was additionally introduced into the solution.

**Table 2:** Calculated values of the average pitting depth test time of 120 days.

Sample	Average Number of Pittings per 1 mm^2^	Average Diameter of Pitting, μ	Pitting Depth, µm
MW-bio	6	13.8	83
MWH-bio	1	26.7	88
MWG-bio	13	18.8	46
MWGS-bio	63	16.1	25

**Table 3:** Parameters of stainless steel in natural water and in the presence of additives.

Sample	E_corr_, mV (s.c.e.)	i_corr_, µA/cm^2^	V_corr_, µm/Year
MW0	−263	0.2334	2.7
MW1-abio (Cl^−^)	−285	0.6930	8.0
MW2-abio (HCO_3_^−^)	−291	0.6268	7.2
MW3-abio (NO_3_^−^)	−272	0.4311	5.0
MW4-abio (SO_4_^2−^)	−320	0.5962	6.9
MW5-abio (S^2−^)	−325	0.4698	5.4
MW6-abio (microbial metabolites)	−236	0.5860	6.8
MW7-abio (S^2−^ + HCO_3_^−^ + microbial metabolites)	−337	1.2127	14.0

## Data Availability

The original contributions presented in the study are included in the article, further inquiries can be directed to the corresponding author.

## Abbreviations

The following abbreviations are used in this manuscript:

RW	Radioactive waste
SNF	Spent nuclear fuel
ESB	Engineered safety barrier
DGR	Deep geological repository
MIC	Microbial-induced corrosion
MLW	Medium-level waste
HLW	High-level waste
